# Carrier Fibers for the Safe Dosage of Nanoparticles in Nanocomposites: Nanomechanical and Thermomechanical Study on Polycarbonate/Boehmite Electrospun Fibers Embedded in Epoxy Resin

**DOI:** 10.3390/nano11061591

**Published:** 2021-06-17

**Authors:** Natalia Cano Murillo, Media Ghasem Zadeh Khorasani, Dorothee Silbernagl, Farnaz Emamverdi, Karen Cacua, Vasile-Dan Hodoroaba, Heinz Sturm

**Affiliations:** 1 Bundesanstalt für Materialforschung und -prüfung (BAM), Unter den Eichen 87, 12205 Berlin, Germany; Media.Ghasem-Zadeh-Khorasani@bam.de (M.G.Z.K.); Dorothee.Silbernagl@bam.de (D.S.); Farnaz.Emamverdi@bam.de (F.E.); Dan.Hodoroaba@bam.de (V.-D.H.); Heinz.Sturm@bam.de (H.S.); 2 Department of Mechanical Engineering and Transport Systems, Technical University of Berlin, 10587 Berlin, Germany; 3 Faculty of Engineering, Instituto Tecnológico Metropolitano (ITM), Cra. 54A #30-01, Medellín 050013, Colombia; karencacua@itm.edu.co

**Keywords:** electrospun nanocomposite fiber, nanomechanical characterization, epoxy nanocomposites

## Abstract

The reinforcing effect of boehmite nanoparticles (BNP) in epoxy resins for fiber composite lightweight construction is related to the formation of a soft but bound interphase between filler and polymer. The interphase is able to dissipate crack propagation energy and consequently increases the fracture toughness of the epoxy resin. Usually, the nanoparticles are dispersed in the resin and then mixed with the hardener to form an applicable mixture to impregnate the fibers. If one wishes to locally increase the fracture toughness at particularly stressed positions of the fiber-reinforced polymer composites (FRPC), this could be done by spraying nanoparticles from a suspension. However, this would entail high costs for removing the nanoparticles from the ambient air. We propose that a fiber fleece containing bound nanoparticles be inserted at exposed locations. For the present proof-of-concept study, an electrospun polycarbonate nonwoven and taurine modified BNP are proposed. After fabrication of suitable PC/EP/BNP composites, the thermomechanical properties were tested by dynamic mechanical analysis (DMA). Comparatively, the local nanomechanical properties such as stiffness and elastic modulus were determined by atomic force microscopy (AFM). An additional investigation of the distribution of the nanoparticles in the epoxy matrix, which is a prerequisite for an effective nanocomposite, is carried out by scanning electron microscopy in transmission mode (TSEM). From the results it can be concluded that the concept of carrier fibers for nanoparticles is viable.

## 1. Introduction

Fiber reinforced polymer composites (FRPC) have been widely used as structural materials in applications where high strength and lightweight is needed. Even though composite polymer materials offer numerous advantages, their inherent low fracture toughness and brittleness are shortcomings that can limit their application. The addition of inorganic fillers led to good results beyond their use as fire retardants, and even at reasonable cost for effective nanosized fillers. For boehmite nanoparticles (BNP), it has been shown that nanoscale effects contribute to the cure chemistry [1] and network formation by forming a soft interphase with the epoxy matrix that successfully dissipates the energy of crack propagation and improves the composite mechanical performance [2,3,4]. It has already been reported that this soft interphase is related to a preferential interaction between the BNP and the hardener of the epoxy system affecting also the crosslink density of the composite [5]. The addition of surface modified boehmite nanoparticles have also been studied as reinforcing nanofillers for epoxy resins [6]; taurine modified boehmite nanoparticles, for example, hardly altered the flowability of the epoxy liquid resin which represents an advantage in the FRPC processability [7]. An improvement in the particle distribution was also found compared the other type of surface modified BNP.

In parallel to the inclusion of inorganic nanoparticles, the addition of thermoplastic, layered plies in FRPC was investigated [8,9]. Thanks to a combination of high yield strength and elongation to fracture, these thermoplastic layers placed between the carbon fiber mats helped to decisively improve the interlaminar fracture toughness, and this with only a thin interlayer of about 16 µm thickness [10]. The use of thermoplastic micro- and nanofibers, which can be produced by electrospinning [11], is also already established. Polyamide 6,6 (Nylon), for example, has been an extended option to be used as a reinforcement material due to its good thermal stability and mechanical strength, proving to increase the interlaminar fracture toughness by 25% [12]. Polyamide electrospun fibers have also been shown to increase the stress at failure during the flexural mechanical tests up to 42%, and to hinder crack propagation according to analysis of the fractured surfaces [13]. Electrospinning has also been used to combine two different polymeric fibers in one single fleece, in order to boost the fracture toughness as an interleaved structure. Here, Polyamide 6,6 and poly (ε-caprolactone) (PCL) were used combined resulting in an increase of the fracture toughness at its initiation stage and during the propagation [14]. PCL non-woven has also been used to heal cracks in composites by using a low melt viscosity PCL to bridge the cracked surfaces [15]. Polyimide nanofibers [16] have been reported to enhance composite toughness. To further optimize interfaces, electrospun polymer fibers were also produced as core-shell structures which, when in contact with a monomer, could integrate chemically at their surface into the newly formed network, i.e., by forming new bonds, to form an effective interphase [17]. Other core–shell fibers, such as poly (methyl methacrylate) (PMMA)–polyacrylonitrile (PAN), PMMA–polystyrene (PS), polybutadiene (PB)–PS, PAN–PMMA, and nylon–PMMA fibers, have been produced by coaxial electrospinning and applied as reinforcement [18,19].

In summary, controlled toughness in FRPC can be achieved by adding nanofillers or thermoplastic nano- and microfibers. To combine both strategies and apply them locally in a component, a thermoplastic fiber with incorporated nanoparticles can be useful if the fiber at least partially releases the nanoparticles into the epoxy matrix. At the same time, the addition of PC microfibers may not have a negative effect. As recently seen in a first finding demonstrated for the same system of PC, resin, and hardener [20], the interaction within this ternary system is complex.

In almost all cases found in literature, electrospun fibers are used for direct amplification of mechanical properties [21]. Therefore, the polymer chosen for fiber production has a melting point higher than the curing temperature for the epoxy resin. The chemical integrity of the nano- and microfibers during the curing process is also an issue when aiming for toughness increase or modulus increase. In this article, on the other hand, the aim is to achieve as much contact as possible between the boehmite and the epoxy resin. In this case, complete dissolution or complete melting of the polycarbonate fiber would be the ideal condition to achieve this.

To the best of our knowledge, the use of nanoparticle-loaded electrospun microfibers to reinforce critical zones in fiber-reinforced plastics (FRP) is new, but the local modification of FRP by such microfibers is not. In 2020, Maccaferri, et al. [22] showed the successful application of highly damping fiber mats made of carboxylated nitrile-butadiene rubber (NBR), which needs to be blended with poly (ε-caprolactone) (PCL) for processing by electrospinning. As in the present work, the effectiveness of the additive NBR is also based on the fact that the thermoplastic carrier material mixes well with the epoxy matrix because the melting temperature of the thermoplastic (PCL Tm 55–65 °C) is exceeded in each case during curing. In this work, PC was chosen not only because of chemical compatibility between PC and the DGEBA resin [20] but also so that there was no danger of the material softening even at low temperatures due to the addition of a low-melting thermoplastic.

In this study, therefore, polycarbonate and taurine modified boehmite nanoparticle fibers were prepared by electrospinning and then embedded in an epoxy matrix. The localized mechanical effect of the embedded fibers was investigated by atomic force microscopy–force distance curves (AFM-FDC) spectroscopy, the spatial distribution of the nanoparticles was explored by scanning electron microscopy in transmission mode (TSEM), and the overall mechanical performance of the composite was estimated by thermomechanical dynamic analysis (DMA).

## 2. Materials and Methods

Bisphenol A Polycarbonate (PC) from Goodfellow, UK with Mw ≈ 49,550 g/mol and Mn ≈ 21,400 as measured by gel permeation chromatography (GPC) was used. As inorganic nanofiller taurine modified aluminum oxide hydroxide (γ-AlOOH) HP14T customized by Sasol, Germany was acquired. The coverage with taurine was ~16% [7] and the boehmite nanoparticles (BNP) had an average primary size of 20 nm. The BNP were mixed in a solution of methylene chloride (CH_2_Cl_2_) and PC with different particle concentrations (10, 15 and 20 wt %).

The EP system used in this study was bisphenol-A-diglycidyl ether (DGEBA) cured with an anhydride curing agent, methyl tetrahydrophtalic acid anhydride (MTHPA), the curing was accelerated by 1-methyl-imidazole (DGEBA, Araldite^®^LY 556, MTHPA, Aradur^®^HY917, and DY070 accelerator Huntsman, Basel, Switzerland). The mixture of epoxy, hardener, and accelerator was 100:90:1 part per weight, respectively. After stirring, the mixtures were cured for 4 h at 80 °C to reach gelation and 4 h at 120 °C for post-curing, as recommended by the manufacturer to obtain a fully cured system.

### 2.1. Electrospinning

To produce the composite fibers, a custom-made electrospinning set up was used. The set up comprised a needle connected to a high voltage supply and a rotating drum as collector, the solutions were electrospun to form fibers at 25 kV and a distance between electrode and collector of 10 cm. The solutions were infused at a constant rate of 4 mL/h using a syringe pump, mats were electrospun at room temperature and kept at a relative humidity of 40%. The non-woven mat was let dry under a fume hood for about 1 h at room temperature before being extracted from the collector and placed in silicon molds with epoxy system for curing as described above.

### 2.2. Energy Dispersive X-ray Spectroscopy (EDX)

Before elemental analysis with EDX, the mats surface was coated with a carbon layer of around 15 nm in order to ensure electrical conductivity. An EDX detector with an active detector area of 30 mm^2^ and an energy resolution of 123 eV (Mn Kα) of the type UltraDry from Thermo Scientific™ (Waltham, MA, USA) coupled with a tungsten thermionic emitter scanning electron microscope (SEM) of type EVO MA 10 (Carl Zeiss Microscopy GmbH, Jena, Germany) were used for the elemental analysis of the fiber mats.

### 2.3. Atomic Force Microscopy (AFM)

All AFM measurements were conducted with an MFP-3D AFM (Asylum Research, Santa Barbara, CA, USA). The AFM probe used was a Mikromasch, HQ: NSC35 (Wetzlar, Germany). The spring constant of the cantilever was determined by a non-invasive thermal noise method to be k_c_ = 9 N/m. The tip radius R = 28 nm was estimated by fitting reference measurements with the Hertz on a glass reference surface. Force–distance curves were recorded with a frequency of 1 Hz and 500 nm apart from each other. The detailed methodology of analyzing FDC curves is described in reference [23]. For the analysis of FDC we used a custom software (SOFA, Berlin, Germany) developed in our group. Hertz theory was applied for the calculation of Young’s moduli from the FDC curves. Due to the necessary larger spacing of the indentations in the measurement of FDC compared to the corresponding topography measurement, the data density must be adjusted to create maps of Young’s module using two-dimensional interpolation. For a statistical approach, histograms of the stiffness maps were plotted. To understand the contribution of each material phase to the overall mechanical properties, the histograms were deconvoluted by multiple Gauss fits using the Fityk 1.3.1 software (Poland) [24].

### 2.4. Transmission Scanning Electron Microscopy (TSEM)

The spatial distribution of BNP in the fiber reinforced sample was investigated with a SEM Zeiss Supra 40 microscope (Zeiss, Oberkochen, Germany) equipped with a high-resolution cathode of Schottky type and conventional Everhart–Thornley (ET) and In-Lens secondary electron (SE) detectors. The nanoparticles as embedded within the fibers in the epoxy matrix were detected using the SEM in the transmission mode (T-SEM) after preparation of the sample as thin, electron-transparent layer [25]. Ultra-microtomed thin sections of the samples of fibers embedded in the epoxy resin, of around 100 nm in thickness, were prepared and deposited on a typical copper TEM grid for evaluation with TSEM, a dedicated sample holder specially developed for TSEM was used.

### 2.5. Thermogravimetric Analysis (TGA)

Compositional analysis by thermogravimetry of the composites was determined according to ASTM E1131-08 method by using a Discovery 550 Thermogravimetric Analyzer (TA Instruments, Delaware, USA) an nitrogen atmosphere at a ramp of 10 °C up to 600 °C and air atmosphere up to 900 °C. Highly volatile matter (ambient temperature to 200 °C), medium volatile matter (200 °C to 600 °C), combustible material (600 °C to 900 °C), and residue were determined.

### 2.6. Confocal Raman Spectroscopy

A Raman microscope (WiTec, Ulm, Germany) in inverse configuration with a stepper-motor driven 63× objective with correction of the transmission thickness was used at a laser wavelength of 488 nm. A glass fiber with 100 µm serves as aperture for measurements of PC/hardener, for measurements at the PC/resin interface an aperture of 25 µm had to be used. The irradiance of the laser was 2.5–2.6 mW. The spectrometer used was a UHTS300 (WiTec, Ulm, Germany) with a grating with 600 grooves per mm. The integration time of the DV 401 spectrometer camera was always 2 s. Resin or hardener was applied to the 72–80 µm thick films of polycarbonate as thick drops with a diameter of a few millimeters. The measuring position was controlled by an optical microscope and set up in such a way that the measurement could begin about 2 min after dripping the fluid. The objective was approached from bottom to top so that the lower air/PC interface was also recorded. This gives a reference position for each measurement, as it was found that the sample moves by a few micrometers when measurements are taken over a few hours.

### 2.7. Dynamic Thermomechanical Analysis

Dynamic thermal mechanical analysis (DMA) were recorded using an ATM3 torsion pendulum (Myrenne, Roetgen, Germany). In this method a clamped sample was loaded within an oscillating pendulum. The sinusoidal shear deformation induced a free oscillation of the pendulum at frequency of 1 Hz with 1° strain. The measured oscillation period and damping were used to calculate the complex modulus G*. The storage and loss moduli, G′ and G″ were determined as a function of temperature. In this work, the temperature was ramped up from 20 to 200 °C with the heating rate of 1 K/min. The measurement was carried with the presence of nitrogen gas in the chamber. DMA measurements were carried out on epoxy samples containing 2 wt % of PC mats, the mats were loaded with nanoparticles in concentration of 0, 10, 15, and 20 wt % BNP content. Sample dimensions were 50 mm × 5 mm × 1 mm. Each sample was measured through first heating up (first run) and cooled down, followed by second heating up (second run). Both heating cycles were further evaluated.

## 3. Results and Discussion

### 3.1. Polycarbonate-Boehmite Electrospun Fibers Morphology

**Figure 1:**
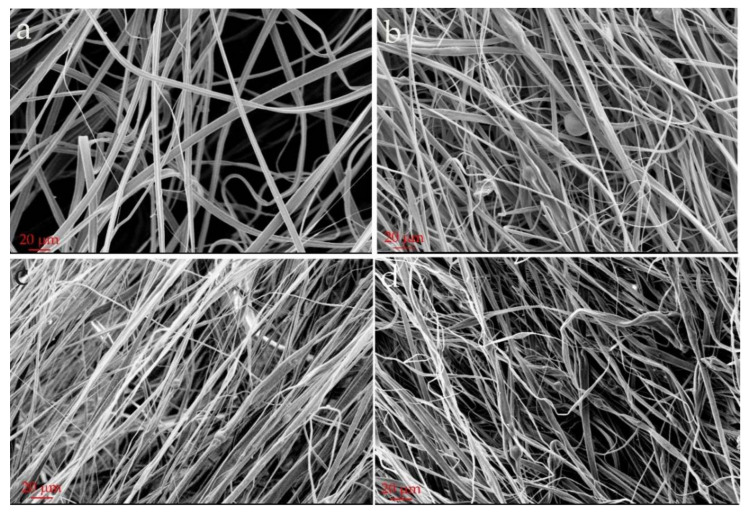
Scanning electron microscopy image of the electrospun fibers: (**a**) Pure polycarbonate; (**b**) Polycarbonate with 10 wt % BNP; (**c**) PC with 15 wt % BNP; (**d**) PC with 20 wt % BNP.

**Figure A1:**
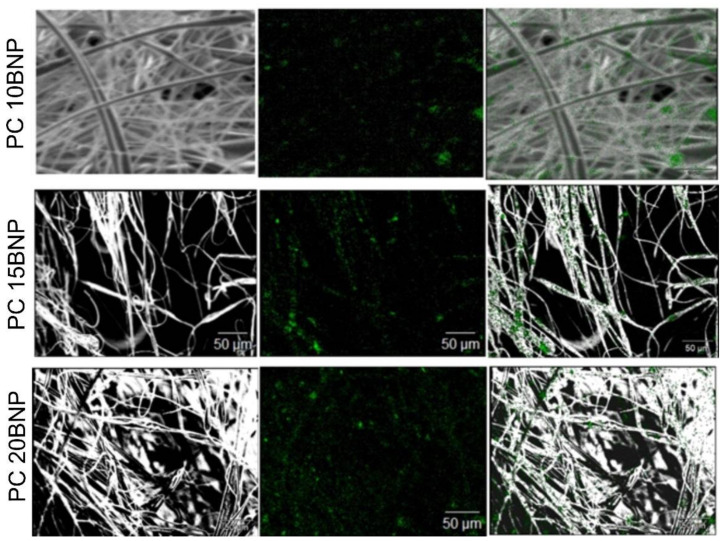
SEM (**Left**), EDX elemental maps showing the Al signal of the fibers (**Middle**) and their superposition (**Right**).

Figure 1 shows the morphology of the obtained electrospun PC fibers and the composite PC/BNP fibers in three different concentrations. The fibers are, in general, ribbon-like. At higher concentrations of BNP, the shape and diameter of the fibers show some variation. The presence of aggregates is indicated by the morphology of fibers containing boehmite, which is expected at this range of BNP concentrations. However, the encapsulation of BNP in the fibers is successful and the aluminum signal along the fibers could be detected with EDX (see Appendix A Figure A1). The fibers also present a porous surface; a detailed study on the structure-properties on the composite fibers will be reported [26]. The porosity on electrospun fibers results from an interplay of the solvent evaporation and the surrounding humidity. Evaporative cooling takes place during solvent drying and consequently water in the surrounding condensates and hence small water droplets locate on the surface of the forming fiber. Once the water on the surface evaporates, the droplets disappear, leaving a space that the polymer could not occupy [27]. Given the ability of BNPs to store water, the condensing water could be bound to the surface and volume of the BNP and stay trapped during the fiber formation. This effect is triggered by the high difference in vapor pressure between the water (bounded to BNP and in the surroundings) and the DCM (at 25 °C water v_p_ = 3.17 kPa and dichloromethane v_p_ = 57.3 kPa) used as polymer solvent [28]. This means that the porosity could have also evolved to internal porous with increasing concentration of BNP, as studied by SAXS measurements of the fibers [26]. The presence of these porosities is a desirable feature since it should increase the probability of the BNPs to be available for the epoxy resin and should shorten the dissolution of the PC.

### 3.2. Epoxy/Neat Polycarbonate Fiber Morphology and Nanomechanical Response

**Figure 2:**
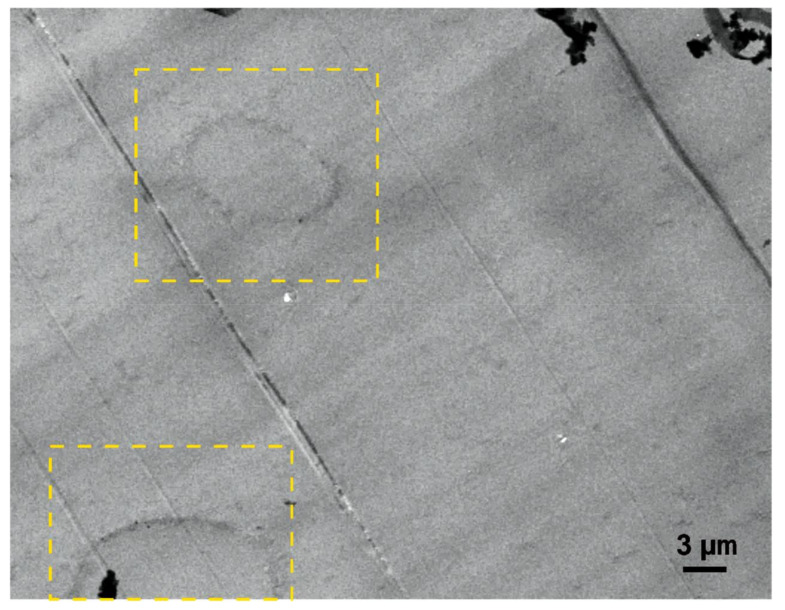
10 kV scanning electron micrograph in transmission mode (TSEM) in different regions of the epoxy composite containing PC neat fibers. The regions in the yellow squares signalize interstices or crossing points of collapsed PC fibers, the rounded regions are filled with epoxy resin. The original image can be found in the Appendix C Figure A3.

The circular features framed in yellow in Figure 2 show a crossing point of collapsed PC fibers after the curing process. The rounded and slightly darker border corresponds to the outer surfaces of former PC fibers (interstice), the interior part is epoxy resin. The higher attenuation of the electrons passing the samples (i.e., lower transmission) should correlate with higher (atom) density, eventually according to a chemical reaction or crystallization (nodular development). The contrast between the epoxy and the PC away from the interface is negligible.

**Figure 3:**
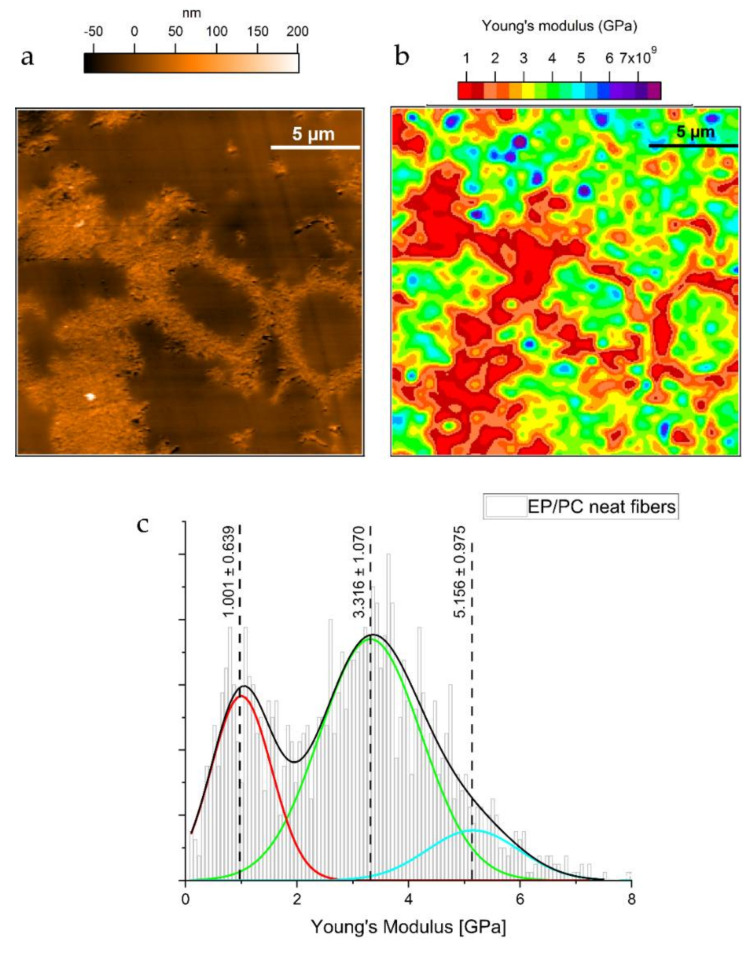
(**a**) AFM Topography map of an area of the composite showing presumably several neat PC fibers; (**b**) Young’s Modulus map of the area shown in (**a**); (**c**) Young’s modulus distribution from the scanned area. The maps were taking from the bulk sample after extracting the ultra-microtomed slices (the Young’s modulus map with 80 × 80 data points is interpolated to match the pixel number of the corresponding topography image, which is 512 × 512 data points). In the deconvoluted signal: red indicates the PC phase, green and light blue correspond to EP phase.

**Figure A2:**
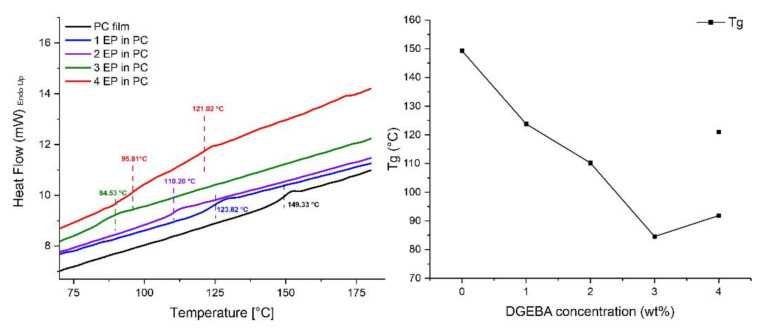
(**Left**) Change on the *Tg* for bar coated films of polycarbonate containing up to 4 wt % of DGEBA. At a concentration of 4 wt % two different *Tg* are detected; (**Right**) The value of the *Tg* is taken as the half of the step in the heat flow signal during the second heating.

In Figure 3a, the AFM topography of the scanned region on the sample of EP/PC neat is shown. The features clearly correspond to one or more PC fibers. The phase separation from the epoxy resin is evident as was already reported for a model system consisting of a layer of PC in contact with an interacting epoxy layer [20], in which a trend of the PC phase to form spherulite-like domains was found. The constitution of spherulites arises from an inherent nodular structure of PC, high chain mobility and the exposure of PC to temperatures between 80 °C and below its *T_g_* ≈ 149 °C. In addition, the mobility of the PC chains in the liquid state is increased by their interaction with the DGEBA monomer. Differential scanning calorimetry measurements (DSC) on solution casted films of PC and 1, 2, and 4 wt % of DGEBA shows a gradual lowering of the measured *T_g_* (see Appendix B Figure A2). A concentration of 4 wt % of the DGEBA monomer in the PC undergoes a phase separation leading to a double appearance of a glass transition. Still, the interaction of PC and the DGEBA monomer entails that at early stages of the curing process some of the PC could be integrated in the growing epoxy network. Nevertheless, as the molecular weight of epoxy increases during the crosslinking reaction, the polycarbonate will have less sites to form linkages which ultimately leads to phase separation. This reaction-phase separation phenomena is frequently found in thermoplastic modified epoxies [29,30].

Thermal analyses of pure electrospun PC fibers confirm that the fibers have a lower glass transition temperature (*Tg*) value. Since the *Tg*, as a measure of the chain mobility, reflects a decrease in the mechanical stiffness of a polymer, it would be expected that the PC phase from the fibers would have a lower Young’s modulus. From the same Young’s modulus map in Figure 3b, some small domains with higher values of Young’s modulus were found between 4.8 and 6.13 GPa. No topographical features corresponding to these areas of high modulus can be detected as can be seen in Figure 3a. This suggests that the epoxy resin shows regions with different network development and consequently different mechanical properties. For this particular epoxy resin a recent study confirms the heterogenous nature of the epoxy network development by exhaustive calorimetric measurements [31]. This is a common characteristic among epoxy resins. The effect on the nodular development has been further investigated by neutron scattering [32], AFM and IR spectroscopy to conclude that epoxy networks tend to exhibit phases with inhomogeneous crosslink density, leading to differences in mechanical properties [33]. Moreover, the differences in mechanical properties might be related to chemical inhomogeneities during the early stages of the curing process as confirmed by AFM-IR studies, where the chemical heterogeneity undergoes nanoscale nodular structure in the epoxy resin with an appearance of densely crosslinked nodules [34].

### 3.3. Epoxy/Polycarbonate/BNP Fiber Morphology and Nanomechanical Response

**Figure 4:**
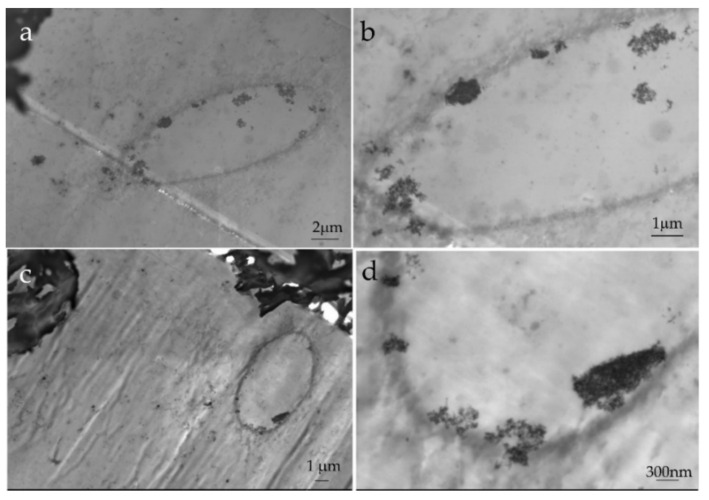
(**a**,**c**) Scanning electron microscopy images in transmission mode (TSEM) of two areas of an interstices between PC fibers inside the epoxy matrix after ultramicrotome sample preparation. Outside the elliptical boundaries of the interstice, the rough structures of partially dissolved PC can be seen (as shown in Figure 3b). (**b**,**d**) Zoomed-in images show the BNP domains at the boundaries between the PC fiber and the epoxy occupying the interstice.

The morphology of the Polycarbonate/BNP electrospun fibers inside the epoxy matrix is displayed in Figure 4. The images (Figure 4a,c) correspond to two different regions of the same ultra-microtomed slice of the sample. The BNP domains are made by TSEM perfectly visible at the boundaries between the epoxy-rich phase in the interstice and the rough structures created by the PC fiber. Interestingly, the boehmite aggregates are located at the boundary between the two polymers as suspected, as if the BNP migrated from the PC phase towards the EP-rich phase. A similar result was already found in our previous work on a model sample, in which the BNP have a trend to agglomerate around the EP region [20]. This tendency could arise from a mobilizing effect of the DGEBA in PC, which along the high curing temperatures, set the conditions for a semi-organized packing of PC chains in which BNP would not take place and are left aside.

**Figure A4:**
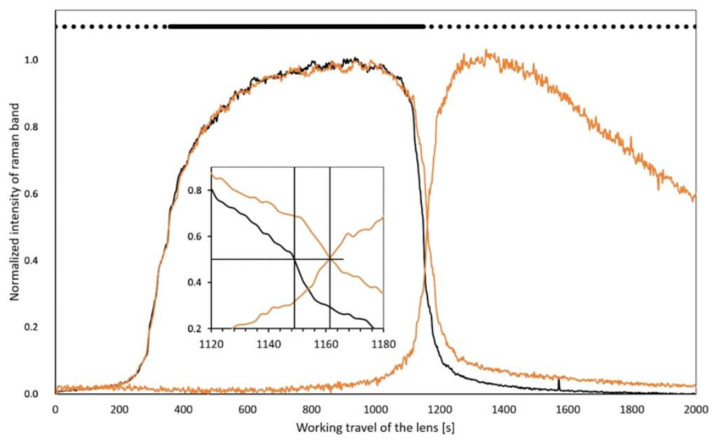
Concentration profile of an interface between a PC bar coated film and a drop of hardener.

**Figure A5:**
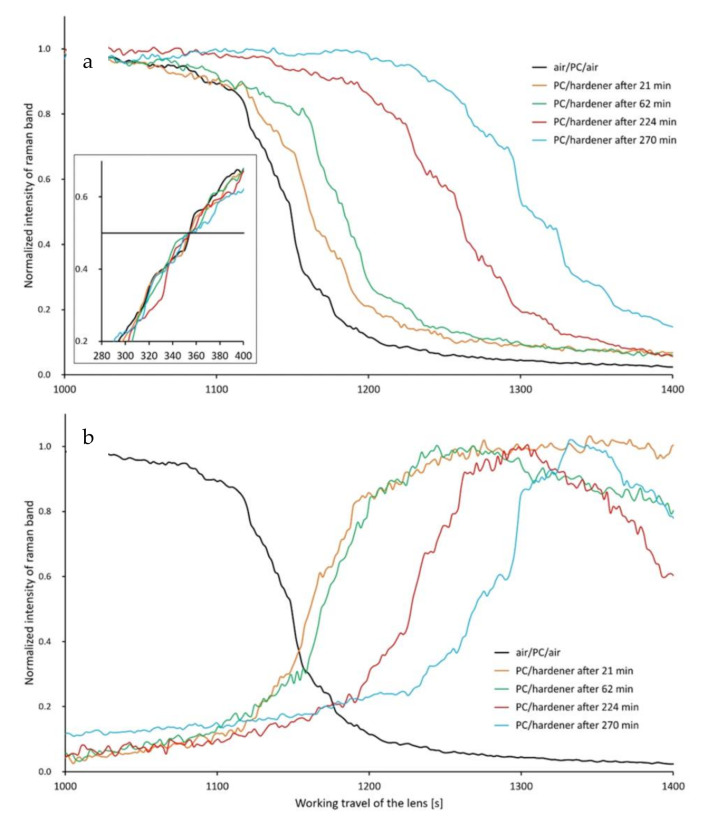
(**a**) Time-dependent analysis of the interface between PC and hardener, here the normalized Raman signal of PC is evaluated using the band at 893 ± 5 cm^−1^; the inset shows the corrected position of the air/PC interface. (**b**) Time dependent analysis of the interface between PC and hardener, here the normalized Raman signal of the hardener is evaluated using the band at 1848 ± 5 cm^−1^.

**Figure A6:**
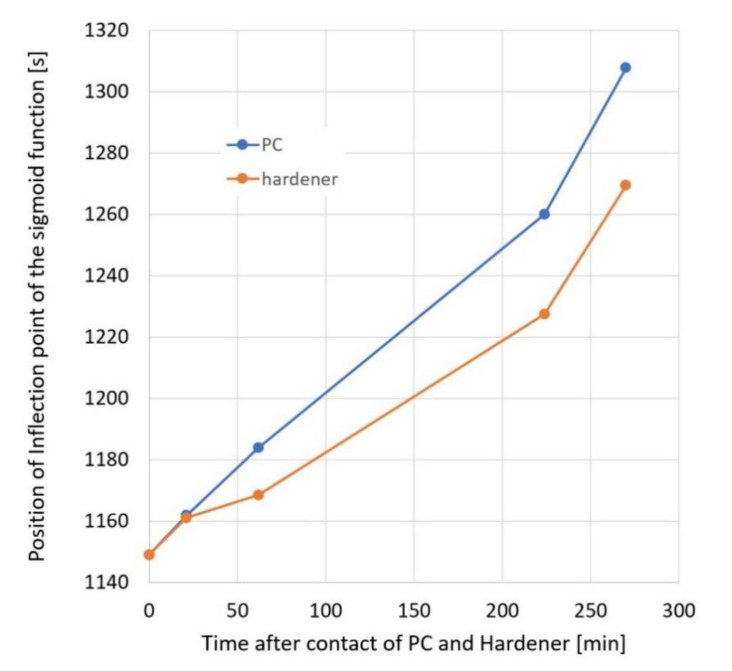
Depending on the contact time of hardener on the PC film, the respective inflection points of the falling (PC) and rising (hardener) sigmoid functions shift. From this it can be concluded that both the hardener penetrates the PC and the PC dissolves in the hardener.

In order to support this hypothesis, two experiments were carried out to demonstrate the change over time of a PC surface in contact with resin respective hardener at room temperature. For this purpose, the interface between the respective liquids and the PC was investigated in a depth scan using confocal Raman spectroscopy. Vibration bands specific to PC, resin, and hardener were used and measured as the interface was traversed. As shown in the Appendix D Figure A4, Figure A5 and Figure A6 the hardener dissolves the PC and a zone of overlap is created, which increases with time. For the PC/resin system, several tests were initially carried out with increasing confocality, as the spatial resolution was initially insufficient. The decreasing intensity and the resulting deterioration in the signal-to-noise ratio could not be compensated by smoothing the data. It must be stated that with the methods used, it was not possible to prove dissolution of the PC or interdiffusion of the epoxy resin.

Even though the map of, e.g., thermal process parameters is only rudimentary so far, it is proven that the boehmite particles are liberated by increasing the mobility of the polycarbonate chains, because the hardener diffuses inside PC even at room temperature.

**Figure 5:**
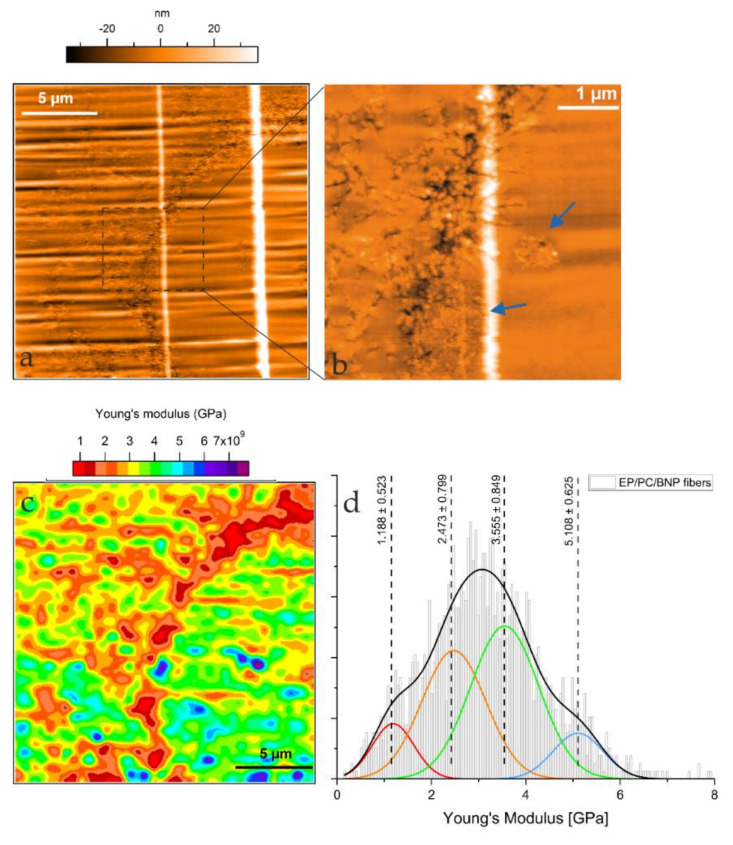
(**a**) Topography of an 80 × 80 µm^2^ area of the composite showing a collapsed PC/BNP fiber inside the epoxy matrix; (**b**) Zoomed-in area showing the topographic features of the area in (**a**), the blue arrows indicate the features which correspond to BNP domains; (**c**) Young’s modulus map of the area shown in (**a**); (**d**) Histogram of the Young’s modulus of the scanned area.

For the purpose of the evaluation of local mechanical properties, only the composite with PC fibers containing 10 wt % of BNP was studied. In Figure 5a,b, the topography of the sample is shown. The scanned area shows a semicircular feature in the topography maps, with structures disseminated all over the epoxy matrix. The mentioned structures show a pattern of arms of small nodules just like in the case of the neat PC fibers inside the epoxy matrix. However, in this case, the growing of such nodules towards spherulite-like domains is suppressed.

A separated ellipsoidal rough structure, of around 1 µm in diameter, is detectable in the EP-rich phase; this domain is indicated with the blue arrows in Figure 5b, which are associated to BNP domains, according to the previous micrographs from EP/BNP systems shown in Figure 4. The BNP domains were expected to show increased stiffness values. This is confirmed by the higher values of the Young’s modulus as small blue regions shown in Figure 5c, where values in the range of 4.48 and 5.73 GPa were calculated from the FDC force spectroscopy. It is important to keep in mind that other blue regions also display high values of the Young’s modulus, yet they cannot be directly associated with BNP domains since no topographical features were found. Interestingly, the EP/PC/BNP system presents less contrast in terms of its components as compared to the EP/PC neat fiber system. The presence of more contributions to the averaged Young’s modulus of the scanned area can be discriminated by the deconvolution of the corresponding histogram (Figure 5d). The contribution to the Young’s modulus of the PC fiber phase is still differentiable with a mean value of 1.188 ± 0.523 GPa in fiber/BNP. A second contribution with a mean Young’s Modulus value of 2.473 ± 0.799 GPa is attributed to a stiffening of the PC domain, this value being in agreement with our previous result for a reinforced PC film in a model sample [20]. The mean Young’s modulus value for the EP phase (3.555 GPa) and a region showing higher values of the Young’s modulus around 5.108 GPa are contributing to the total mechanical response that seems to be more uniform when compared to the EP/PC fiber neat system. The reason for a more uniform localized mechanical behavior could be that the water stored inside the fibers and is bounded to BNP promotes chain scission of the PC during the curing process of the resin; this is plausible given the acceleration of thermal decomposition that the PC fibers suffer with concentrations of BNP higher than 5 wt % [26]. The existence of interphases could also be a reason for this characteristic response; interphases between BNP and PC and PC and EP were studied previously on our group [35], from which attractive forces (*F_attr_*) measured by Intermodulation AFM (ImAFM) account for mixed behavior of the composite in which the BNP domains remained surrounded by PC, and the PC phase was strongly permeated by EP. Moreover, the plasticizing effect that DGEBA has on PC and the diffusion of hardener molecules into the PC phase change the distribution of species in the network. From the Young’s modulus map, it is possible to infer that the heterogeneity of the ternary system achieves a synergistic response of localized mechanical properties.

### 3.4. Dynamic Mechanical Thermal Analysis (DMA) of the Composites

#### 3.4.1. Storage Modulus G′

**Figure 6:**
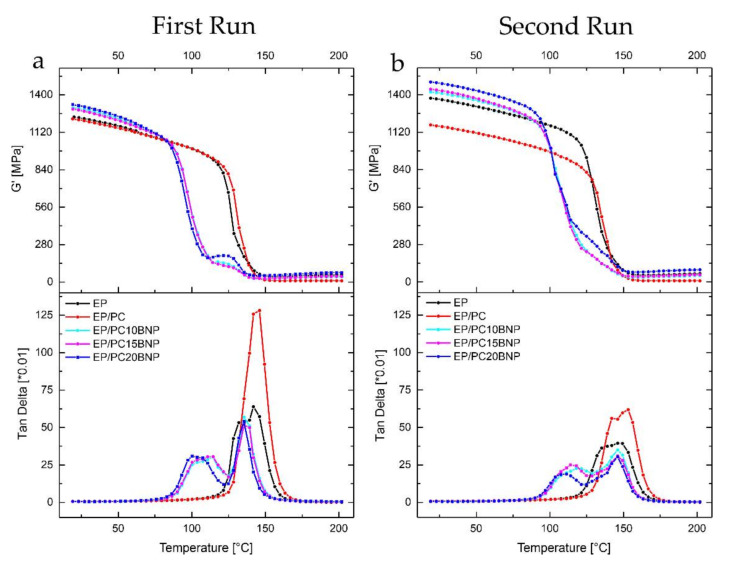
(**a**) Storage modulus G′ and loss factor *Tan δ* during the first run for neat Epoxy and the EP/PC/BNP fiber composites; (**b**) Storage and loss factor *Tan δ* during the second run for neat Epoxy and the EP/PC/BNP fiber composites.

**Figure A8:**
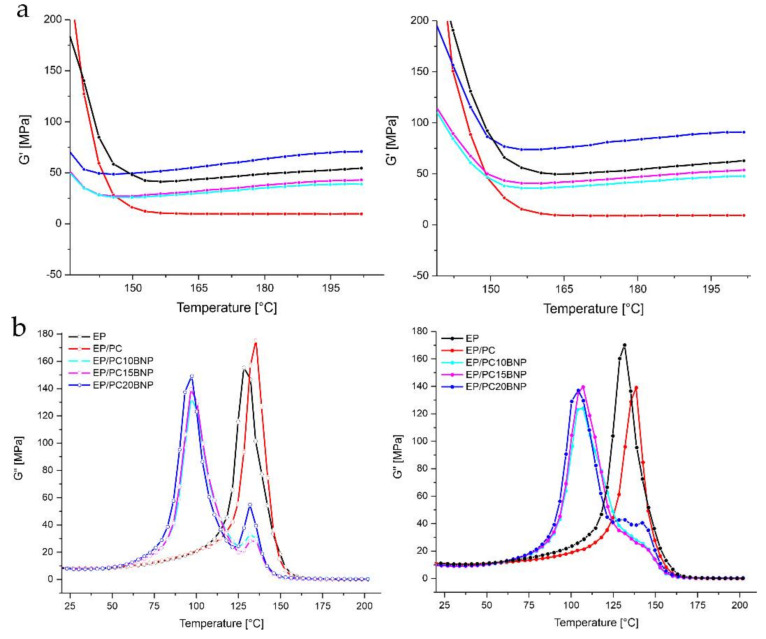
(**a**) Rubbery plateau of the storage modulus G′. Left corresponds to the first heating run and, on the right, the second heating run; (**b**) Loss Modulus G″ of the composites. Left corresponds to the first heating run and, on the right, the second heating run.

Figure 6a shows the storage modulus G′, and *Tan δ* of the neat EP, EP/PC fibers, and the EP/PC/BNP composites during the first and second heating cycles. The corresponding rubbery plateau of the G′ and the loss modulus G″ are shown in the Appendix F Figure A8. The storage modulus G′, roughly speaking, quantifies the flexural or tensile strength of a material. An increase of the G′ at room temperature from the samples containing polycarbonate fibers with 10, 15, and 20 wt % of BNP can be noticed in both heating cycles and are summarized in Table 1. The addition of neat PC fibers to the epoxy matrix does not show a significant change in the storage modulus at room temperature, while the inclusion of the BNP in the PC fibers raises the G′ in the glassy region of the whole composite following a linear trend.

This suggests that the BNP carried by the PC fibers are accessible to cause a stiffening effect in the epoxy system as previously reported [36,37]. This hypothesis is reasonable, given that an increase of the stiffness in the glassy state of the EP/BNP composite is achievable with increasing concentration of BNP, as demonstrated by DMA analysis [5]. Nevertheless, with the addition of taurine modified BNP to an epoxy matrix, the flexural modulus and the bending strength only improved after reaching concentration as high as 5 wt % [7]. In our case, the concentration of BNP within the EP/PC composite was less than 0.4 wt % for all the samples. The char yield from TGA measurements, which is directly associated with BNP content in the composites, can be found in the Appendix E Table A1. The reinforcing effect of these kind of modified BNP at this low concentration is as remarkable as unexpected and it is inferred to be only possible by a synergistic effect involving the three composite phases.

The sudden raise of G′ in the region between 110.7 °C and 138.9 °C for all the composites with BNP is associated with crosslinking induced vitrification. This means that the network reached a higher degree of crosslinking and a higher G′ modulus; however, the composite gets into devitrification and reaches its *T_g_* [38]. The implications of the crosslinking induced vitrification in the EP/PC/BNP composites will be discussed in the section where *Tan δ* is analyzed.

During the second heating, all composites showed an increased G′ at the glassy state. Neat EP showed an increase of more than 200 MPa in comparison to the first heating. It was already reported by Khorasani et al. [5] that the heterogeneous structure of this particular epoxy system can be due to the existence of segments which are not fully reacted. In this case it was expected that exposure to high temperatures during the first run caused an additional post-curing and consequently the matrix system became more homogeneous with higher crosslinking density; this yielded a higher storage modulus in the glassy state. The total opposite behavior was exhibited by the EP/PC fiber composite: G′ had a slight decrease after the first run, and it was assumed that the presence of PC hindered the crosslink density and rendered a more ductile composite endowed with higher flexibility in the polymer chains and higher molecular weight between crosslinks [39].

Taking a look at the rubbery plateau of the storage modulus (Figure A8a in Appendix F) of all the composites, it can be inferred, qualitatively, that the inclusion of the neat PC fibers in the epoxy matrix causes a dramatically decrease of the crosslink density compared to the pure EP and the EP/PC/BNP composites. Introducing PC fibers to the system leads to an altered crosslinking reaction within the epoxy matrix, where some regions remain uncured. During the second run, G′ in its rubbery plateau shows that the crosslink density did not increase.

By increasing the BNP concentration in composites, a trend towards higher crosslink density was observed. At the concentration of 20 wt % BNP in the fibers, G′ in rubbery plateau was slightly higher than in the neat EP, thus, it can be assumed that BNP were acting as crosslinking centers, inducing tight entanglements in their surroundings.

#### 3.4.2. *Tan δ* Analysis

The *Tan δ* peaks of all the composites are shown in Figure 6a in the first heating and Figure 6b for second heating cycle. Neat EP exhibited a bimodal peak as would be expected given its heterogeneous nature [5,31]; during the second heating cycle, the height of the *Tan δ* decreased, and became broader, which we interpret as a higher crosslink density in which the heterogeneous nature of the network persists.

Examining the *Tan δ* peak of the EP/PC composite, a single peak between the EP and neat PC fibers was detected; this means that, macroscopically, the composite behaved as a single-phase material. Nevertheless, it is important to notice that, particularly *Tan δ*, showed a slightly higher temperature at its maxima and that the height of the peak was considerably larger compared to that corresponding to pure EP. From studies of blends of EP and PC, it is known that concentrations of up to 6 wt % of PC in EP can lead to an slight increase in the *T_g_* of the blend [40]. This effect was associated to a reduction of the activation energy of the curing reaction in the presence of PC; as a consequence an acceleration of the cure reaction took place and produced a higher conversion of the epoxy. However, as the height of the *Tan δ* peak is related to segmental mobility of the network [41], the increase in the segmental mobility is believed to be related partially to de-bonding and re-bonding of low energy hydrogen bonds formed between the carbonyl group in PC and the hydroxyl group from the DGEBA monomer. These kind of bonds would contribute with a higher number of moieties sensitive to heating and stresses [42], and simultaneously, would preserve the composite storage modulus, as hydrogen bonding between PC and EP acts as physical crosslinks causing higher packing density [40]. PC phase then precipitates and tends to organize forming spherulites (as shown in Figure 3a). We deduce from this that its molecular weight has not been affected and that under the used curing conditions and concentration (2 wt %) it can co-exist with the epoxy continuous phase, increasing its damping capacity by the presence of hydrogen bonds [30,43]. Still, a separated PC phase of high molecular weight might have an effect of hindering the overall crosslink density of the composite by decreasing the concentration of crosslink points. During the second heating, *Tan δ* peak of the EP/PC composite was reduced in height and broadened following the same trend as pure EP. The reduction in height means that the first heating run also induced a higher packing, but still to a lesser degree compared to neat EP.

The behavior of the EP/PC/BNP *Tan δ* peaking during the first heating showed two well defined peaks that account for the existence of two phases. Examining the *Tan δ* peaks, the phase separation from the composites was evident. There are several possible factors contributing to diversity on the *Tan δ* response:

One of these factors is the crosslinking induced vitrification, which presents its corresponding *Tan δ* maximum at the temperature of the crosslinking process. This maximum is known as *T_gg_,* which is the glass transition temperature of a reactive system at its gelation point [44]. This first maximum is related to the last stages of the formation of an epoxy network which ends up exhibiting a different nature.

A second factor is the degradation of PC caused by the dehydration of the BNP. An acceleration on the rate of thermal degradation was confirmed by TGA analysis on the PC/BNP fibers mat [26]; such dehydration of the BNP during curing of epoxy can cause PC hydrolysis and consequently chain scission This means that the PC phase will consist as well in smaller chains and oligomers. The PC chains can suffer further scission by the transesterification reactions with the DGEBA [45]. Some of the formed shorter chains might be integrated in the epoxy network, but with an EP phase continuously reticulating, the PC chains will be segregated and induce dilution effects. These shorter chains result acting as plasticizers of the epoxy matrix, lowering the *T_g_* of the composite. The PC-BNP phase might be able to have higher segmental mobility and capability of energy dissipation. Normally, dilution effects of thermoplastic phases in thermosetting matrices will render ductile blends, which ultimately manifests as a reduction in storage modulus [46]. In the case of the EP/PC/BNP composites, it appears that if such reduction in storage modulus takes place, then it is compensated by the mechanical properties of the nanoparticles. One last factor to consider is the alteration of the crosslink density in the epoxy matrix caused by the BNP. This factor would also comprise that the BNP are available from the PC fiber phase towards the EP phase [36], the BNP are prone to react with the anhydride hardener of the epoxy resin system and change the construction of the epoxy network in a way that can act as crosslink centers. The location of the BNP in between the PC fiber and the EP matrix, as seen in Figure 4 and Figure 5, is an example of this third factor.

In Table 2 and Table 3, the positions of the *Tan* *δ* peaks, the full width of the half maximum (FWHM), and the peak heights corresponding to all the samples during the first heating and second heating respectively are summarized. The *Tan* *δ* signal was deconvoluted for all samples resulting, in most cases, more than one *Tan* *δ* peak.

During the second run, *Tan* *δ* shows the heterogenous nature of the BNP containing composites. The height of *Tan* *δ* is associated with segmental mobility of network and its broadening is related to the distribution of species able to dissipate energy [41]. The height of *Tan* *δ* is decreased for all samples interpreted in less segmental mobility with diversity of damping species. Decrease in segmental mobility confirms a post curing effect, where the number of free chain ends is reduced after exposure to a second heating cycle.

## 4. Conclusions

Electrospun polycarbonate fibers were studied in this work as a carrier structure for the local reinforcement of an epoxy matrix. The macroscopic, temperature dependent mechanical properties were investigated by DMA. Nanomechanical properties of the composites were examined by AFM-FDC force spectroscopy from which the local Young’s modulus was calculated. It was shown that the presence of neat PC fibers reduced the crosslink density of the epoxy matrix but increased its damping capacity. Mapping the local Young’s modulus on the sub-microscale of EP/PC neat fibers composite shows a phase separated system, in which PC tend to develop spherulitic structures and precipitate from the EP matrix. However, filling the PC fibers with BNP changes the interaction of carrier fibers and EP matrix significantly. For one, the nanocomposite PC fibers show a porous structure which promotes the interaction of the carrier fiber with the matrix. On the submicroscale, the ternary composite shows contributions to the overall mechanical behavior from their individual PC and EP phases along with intermediate phases which correspond to a BNP-reinforced PC phase. Interestingly, the whole composite has a more uniform mechanical response when compared with the neat EP/PC fiber system. The dynamic thermomechanical analysis shows a trend towards higher storage modulus at room temperature with increasing content of the nanoparticles in the PC fibers. The effect is caused by a combination of different aspects: (i) plasticizing effect on PC by the DGEBA monomer, (ii) the ability of the anhydride hardener to diffuse inside the PC phase as was proven by Raman spectroscopy, (iii) chain scission of the PC induced by dehydration of BNP during the curing of the resin, and (iv) ability of the BNP to react with the hardener. The EP/PC/BNP system exhibits not only an increase in the Young’s modulus and a higher storage modulus with higher BNP content but also renders a more ductile epoxy matrix by the addition of the PC fibers. The ternary system works in a synergistic way by balancing these two effects. From this, we conclude that electrospun PC/BNP are good candidate structures for the release of nanoparticles in an epoxy matrix achieving enhanced mechanical properties while allowing the control of local reinforcement with the inclusion of nanoparticles.

## Figures and Tables

**Table 1:** Storage modulus G′ at 25 °C of all composites in the first and second heating.

Sample	First Heating	Second Heating
Epoxy	1203.98	1471.04
EP PC neat	1220.39	1173.51
EPPC 10BNP	1303.05	1421.28
EPPC 15BNP	1294.67	1441.42
EPPC 20BNP	1327.03	1496.02

**Table 2:** *Tan δ* First Run.

Sample	Peak Position T_g_ (°C)			Width of Half Height (C°)			Peak Height (a.u)		
	*Tg_1_*	*Tg_2_*	*Tg_3_*	*FWHM_1_*	*FWHM_2_*	*FWHM_3_*	*Height_1_*	*Height_2_*	*Height_3_*
EP	--	130.59	143.18	--	10.27	15.32	--	40.17	60.98
EP/PC fibers	--	--	143.51	--	--	16.817	--	--	128.19
EP/PC10BNP	109.50	--	137.06	27.30	--	12.88	29.43	--	52.40
EP/PC15BNP	101.37	115.64	136.95	16.62	16.34	12.81	22.96	24.97	50.89
EP/PC20BNP	97.33	105.44	135.14	7.2	23.80	11.91	7.03	28.73	49.26

**Table 3:** *Tan δ* Second Run.

Sample	Peak Position T_g_ (°C)			Width of Half Height (C°)			Peak Height (a.u)		
	*Tg_1_*	*Tg_2_*	*Tg_3_*	*FWHM_1_*	*FWHM_2_*	*FWHM_3_*	*Height_1_*	*Height_2_*	*Height_3_*
EP	--	135.52	150.32	--	17.21	15.75	--	32.25	32.96
EP/PC fibers	--	141.08	153.79	--	13.69	14.65	--	44.15	54.43
EP/PC10BNP	--	119.9	146.44	--	34.74	15.42	--	22.35	29.48
EP/PC15BNP	114.68	133.32	146.41	25.50	9.98	15.52	24.25	9.79	29.71
EP/PC20BNP	109.95	131.40	145.69	23.16	11.89	14.49	18.62	11.48	28.42

**Figure A3:**
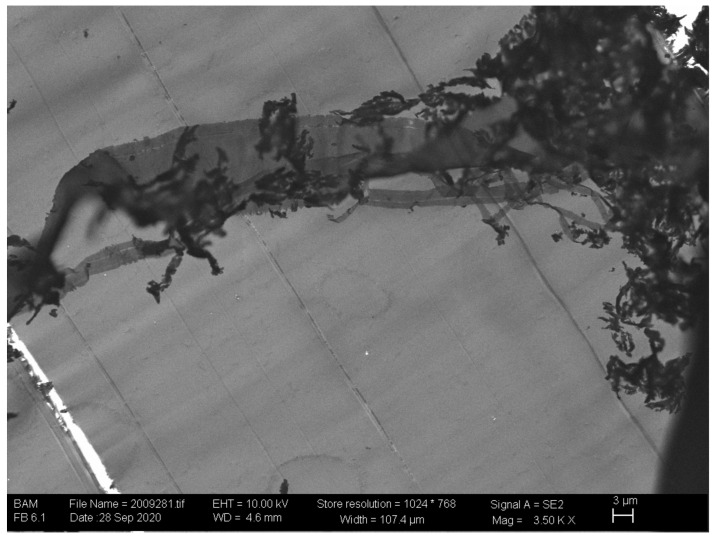
Scanning electron image in transmission mode (TSEM) of the epoxy composite with crossing collapsed PC neat fibers, which appear as slightly darker ellipsoidal structures. The darker structures are shreds from the sample resulting from the ultramicrotome cut.

**Figure A7:**
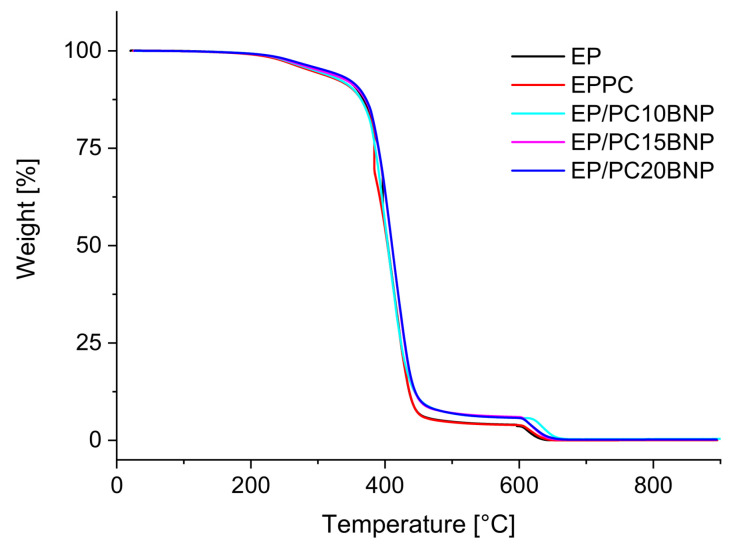
Thermogravimetric analysis on the epoxy, epoxy/polycarbonate, and epoxy/polycarbonate/BNP composites.

## Data Availability

The data presented in this study are available on request from the corresponding author.

## Appendix A

The fiber samples were examinated by EDX to detect the aluminum signal along the fibers.

## Appendix D

**Examination of the polycarbonate/hardener interface by Raman spectroscopy.** The lens is approached from bottom to top, so that the lower air/PC interface was also captured. Complete spectra were recorded all 2 s, integration time 2 s. The black line shows the air/PC/air interface, plotted is the normalized Raman band at 893 ± 5 cm^−1^ against the displacement of the lens, measured in seconds. The right side in Figure A4 shows the PC/hardener interface (hardener at 1848 ± 5 cm^−1^). The inflection points of the sigmoid function are regarded as position points for the transition. The shape of the sigmoid changes, therefore a conversion of the time axis into local positions is not possible. The shift is coupled to a change in the local refractive index (air to hardener), which unpredictably changes both the coupling of the light and the capture cross-section for the Raman photons in the confocal setup. The measurement started 120 s after the hardener was dropped on the surface (t = 0 s). The insets in Figure A4 show a zoom into the region of the PC/hardener interface. The initial thickness of the PC sample is 72 µm.

The times in the legend refer to the time difference between the PC/hardener contact and the time span when the interface is reached. The inset in a show the manually corrected position for the always co-determined air/PC interface as an always fixed position reference. The data of the black curve in Figure A5 is identical to those of Figure A4.

## Appendix E

Figure A7 shows the thermogravimetric analysis on the epoxy, epoxy/polycarbonate, and epoxy/polycarbonate/BNP composites and Table A1 shows the values of different components according to ASTM E1131-08, the onset temperature of degradation (5% mass loss) and the maximum temperature during degradation of medium volatile matter. For high volatile matter, medium volatile, combustible, and char were similar for all samples. Char yield percentage is associated to BNP filler and was superior in EPPC 20 BNP, confirming the higher concentration in this composite. The onset temperature of degradation (5% of mass loss) decreased for EP/EPPC/BNP, which is associated to the start of EPPC degradation. However, after this event, the maximum temperature was increased indicating a higher thermal stability of the composites compared to EP and EPPC as a consequence of BNP nanoparticles presence.

**Table A1:** Compositional analysis by ASTM E1131, onset of degradation temperature and maximum degradation temperature.

Component [%]	EP	EPPC	EPPC 10BNP	EPPC 15BNP	EPPC 20BNP
High volatile	0.74	0.92	1.29	0.97	0.83
Medium volatile	95.05	94.95	92.04	93.51	93.06
Combustible	4.20	4.11	6.31	5.37	5.83
Char	0.05	0.03	0.38	0.16	0.29
Temperature 5% mass loss	306.60	288.30	253.10	286.27	293.77
Maximum temperature [°C]	396.90	384.42	400.40	409.76	410.87
